# Functional modulation of phosphodiesterase-6 by calcium in mouse rod photoreceptors

**DOI:** 10.1038/s41598-021-88140-8

**Published:** 2021-04-26

**Authors:** Teemu Turunen, Ari Koskelainen

**Affiliations:** Department of Neuroscience and Biomedical Engineering, Aalto University School of Science, P.O. Box 12200, 00076 Aalto, Finland

**Keywords:** Cellular neuroscience, Retina, Molecular neuroscience

## Abstract

Phosphodiesterase-6 (PDE6) is a key protein in the G-protein cascade converting photon information to bioelectrical signals in vertebrate photoreceptor cells. Here, we demonstrate that PDE6 is regulated by calcium, contrary to the common view that PDE1 is the unique PDE class whose activity is modulated by intracellular Ca^2+^. To broaden the operating range of photoreceptors, mammalian rod photoresponse recovery is accelerated mainly by two calcium sensor proteins: recoverin, modulating the lifetime of activated rhodopsin, and guanylate cyclase-activating proteins (GCAPs), regulating the cGMP synthesis. We found that decreasing rod intracellular Ca^2+^ concentration accelerates the flash response recovery and increases the basal PDE6 activity (*β*_*dark*_) maximally by ~ 30% when recording local electroretinography across the rod outer segment layer from GCAPs^−/−^ recoverin^−/−^ mice. Our modeling shows that a similar elevation in *β*_*dark*_ can fully explain the observed acceleration of flash response recovery in low Ca^2+^. Additionally, a reduction of the free Ca^2+^ in GCAPs^−/−^ recoverin^−/−^ rods shifted the inhibition constants of competitive PDE inhibitor 3-isobutyl-1-methylxanthine (IBMX) against the thermally activated and light-activated forms of PDE6 to opposite directions, indicating a complex interaction between IBMX, PDE6, and calcium. The discovered regulation of PDE6 is a previously unknown mechanism in the Ca^2+^-mediated modulation of rod light sensitivity.

## Introduction

Cyclic nucleotide phosphodiesterases (PDEs) regulate numerous processes essential in health and disease^1^. These molecules hydrolyze cyclic AMP and cyclic GMP, second messenger molecules involved in a vast number of signaling pathways, including gene regulation, cell proliferation and differentiation, metabolism, immune function, memory, and visual transduction^2,3^. PDEs, together with the cAMP and cGMP synthetizing cyclases, regulate the intracellular cyclic nucleotide concentrations. Therefore, the modulation of the activity of different PDEs is among the key therapeutic targets in humans and animals^4,5^.

Our visual system can function in different illuminations covering a 10^12^-fold range^6^. This requires efficient light adaptation mechanisms in various levels of visual perception, which in the photoreceptor level are mostly mediated by controlling intracellular Ca^2+^ concentration. Here we investigate calcium-dependent modulation of PDE6, an enzyme that hydrolyzes cGMP and mediates the conversion of photon absorption to an electrical signal in the phototransduction cascade, taking place in the outer segments of vertebrate photoreceptor cells. In rod photoreceptors, photon-activated rhodopsin sequentially activates several G-proteins, transducins. The activated transducins bind to PDE6 molecules and dislocate the inhibitory PDE6γ-subunits covering the catalytic sites. This converts PDE6 molecules from their basally active form, where the enzyme activity is set by spontaneous thermal activations, to light-activated form, which hydrolyzes cGMP at nearly diffusion-limited rate^7^. PDE6 activation causes a rapid drop in the cGMP concentration, leading to the closure of cyclic nucleotide-gated (CNG) channels in the rod outer segment plasma membrane, reduction of the circulating “light-sensitive” current, and hyperpolarization of the cell (for reviews, see, e.g.^8,9^).

The closure of CNG channels upon light absorption diminishes the inflow of calcium into the photoreceptor cells, while the continuing extrusion of calcium by the Na^+^/Ca^2+^K^+^ (NCKX) exchangers located in the outer segment plasma membrane leads to a rapid decrease in the cytoplasmic calcium concentration. With strong light stimuli, the intracellular calcium level can drop by more than tenfold in mouse rods^10^. The decrease in Ca^2+^ activates fast light adaptation mechanisms through calcium sensor proteins and accelerates the photoresponse recovery. Guanylate cyclase-activating proteins (GCAPs) augments the cGMP synthesis by the activation of guanylate cyclase^11^. Recoverin controls the rhodopsin kinase affinity to rhodopsin and modulates the lifetime of light-activated rhodopsin^12–14^ and possibly light-activated PDE6^13,15,16^. Calmodulin can modulate the affinity of cGMP to CNG channels^17^, but this mechanism has been regarded as negligible for mammalian rod light adaptation^18^. Further, we, together with others, demonstrated that in addition to GCAPs and recoverin, there exists an unknown calcium-mediated light adaptation mechanism in mouse rods^16^.

In this study, we recorded local electroretinography across the rod outer segment layer (LERG-OS) from isolated mouse retinas with microelectrodes. We demonstrate that lowering of the extracellular calcium concentration reduces the light-sensitivity of rods by accelerating the photoresponse recovery in GCAPs^−/−^ recoverin^−/−^ background. Using the cGMP clamp paradigm^19^ and the modeling of dim flash responses, we found that a drop in calcium concentration can cause ~ 30% increase in the basal PDE6 activity. In addition, we found that the decrease in the extracellular calcium concentration modulates the inhibition efficacy of the non-selective competitive inhibitor, 3-isobutyl-1-methylxanthine (IBMX), for PDE6, and surprisingly, this modulation shifts the inhibition constants against the light-activated and basally activated forms of PDE6 to opposite directions. This suggests a complex interaction between IBMX, PDE6 subunits, and calcium.

## Materials and methods

### Experimental

#### Ethical approval

The use and handling of the animals were in accordance with the Finnish Act on the Protection of Animals Used for Scientific and Educational Purposes (497/2013) and the Government Decree on the Protection of Animals Used for Scientific and Educational Purposes (564/2013). The study was carried out in compliance with the ARRIVE guidelines.

#### Animals and preparation

GCAPs recoverin double knockout (DKO) mice of both sexes, derived from GCAPs^−/−^ and recoverin^−/−^ mice^11^ kindly provided by Dr. Jeannie Chen (University of Southern California), were used in this study. The mice were in 12/12 h dark/light cycle, and they were let to dark-adapt overnight before the experiment. The mice were euthanized by CO_2_ inhalation and cervical dislocation. The eyes were enucleated, and a small incision was made along the equator of the eyes. The eyes were bisected by enlarging the incision with micro scissors, and the isolated eyecups were placed to cooled nutrition solution. One eyecup was stored at + 7 °C in the nutrition solution in a light-tight container to be used later on the same day. The retina was removed from the eyecup with forceps and micro scissors under a microscope, and the whole retina was placed in the recording chamber inside a light-protective Faraday cage. All procedures were performed under dim red light.

#### Recording chamber, the recordings, and perfusion

A recording chamber used in the experiments allowed light stimulation and perfusion of the retina and had a clear passage for microelectrodes^19^. The retina was placed on a filter paper photoreceptor side upwards. Local electroretinography was recorded across the rod outer segment layer (LERG-OS). The recording electrode (tip diameter 2–5 µm) was passed into the depth of ~ 25 µm in the retina, and the reference electrode (tip $\emptyset$ ~ 30 µm) was brought to the retinal surface. The surface of the retina was identified from the voltage shift that took place when the electrode penetrated the surface of the retina. Simultaneous transretinal ERG recordings were conducted with macroelectrodes on both sides of the retina to monitor the condition of the retina.

During the experiments, the nutrition solution filled the open chamber, and the laminar flow of the solution perfused the photoreceptor side of the retina at a constant rate (~ 3 ml/min). The composition of the nutrition solution was (mM): Na^+^, 133.4; K^+^, 3.3; Mg^2+^, 2.0; Ca^2+^, 1.0; Cl^−^, 143.2; glucose, 10.0; EDTA, 0.01; HEPES, 12.0, adjusted to pH 7.5 with 5.8 mM NaOH. The viability of the retina was improved by adding 0.72 mg/ml Leibovitz culture medium L-15 to the solution. For simultaneous TERG recordings, the synaptic transmission to bipolar cells was blocked by adding 2 mM sodium aspartate^20^, and 50 µM BaCl_2_ was added to the nutrition solution to abolish the glial component arising from Müller cells^20,21^. These substances did not affect the LERG-OS signal with the used concentrations^22^. 3-isobutyl-1-methylxanthine (IBMX), a cell-permeant competitive PDE6 inhibitor, was added to the nutrition solution in concentrations of 5, 10, 20, and 40 µM. All chemicals were purchased from Merck Group / Sigma-Aldrich (Darmstadt, Germany).

The calcium-dependent mechanisms in rod photoreceptors were manipulated by decreasing the Ca^2+^ concentration to a very low level. In the low Ca^2+^ solution, the total calcium concentration was adjusted to 100 µM (including 66 µM calcium from 0.72 mg/ml L-15 supplement), and free calcium concentration of the solution was set to ~ 20 nM with 3.4 mM EGTA, calculated with the “EGTA calculator”^16,23^. The pH of this solution was adjusted to 7.5 with NaOH.

The recording chamber was placed on top of a heat exchanger, the temperature of which was controlled with a water circulating heating bath (LTD6G; Grant Instruments Ltd, Shepreth, Royston, UK). All recordings were conducted at 37 ± 1 °C. The temperature in the recording chamber was continuously monitored with a calibrated thermistor (30K6A309I; BetaTHERM; Measurement Specialties, Inc., Hampton, VA, USA). The perfusing solution was connected to the signal ground with a 4.7 µF capacitor to reduce high-frequency noise.

#### Light stimulation

Light stimulation was accomplished with a LED (Luxeon Rebel LXML-PM01-0100, λ_max_ = 532 nm; Lumileds, Amsterdam, Netherlands). The homogeneity of the full field stimulus beam on the retina was verified with a camera-based beam profiler (Model SP503U; Spiricon Laser Beam Diagnostics, Ophir-Spiricon Inc., Logan, UT, USA). 1 ms light flashes were used to stimulate the retina. The absolute light intensity incident on the retina was measured with a calibrated photodiode (FDS100-cal; Thorlabs GmbH, Newton, NJ, USA). The amounts of rhodopsin photoisomerizations in rods (R*rod^−1^ or R*rod^−1^ s^−1^) caused by the stimuli were calculated based on the rod outer segments dimensions (Ø = 1.4 µm, l = 24 µm), the LED emission spectrum, the photodiode spectral sensitivity curve, and the pigment template^24^ as described in^25^.

In addition, a closed-loop proportional-integral-derivative (PID) controlled feedback from the recorded LERG-OS voltage signal to the light source was used in the cGMP clamp procedure to keep the recorded signal constant by adjusting the background light level after the introduction of PDE inhibitor IBMX to the retina. The closed-loop light control was accomplished digitally in LabVIEW software.

#### Data acquisition

Data acquisition and LED controls were handled with a data acquisition card (PCIe-6351; National Instruments, Austin, TX, USA) and custom-made LabVIEW software. The recorded DC signals (LERG-OS and transretinal ERG) were sampled at 1000 Hz with a voltage resolution of 15 nV. The signals were first low-pass filtered with a cut-off frequency of f_c_ = 500 Hz (8-pole Bessel filter) and afterward digitally filtered with f_c_ = 100 Hz.

### Experimental design and statistical analyses

The calcium-mediated control of GCAPs^−/−^ recoverin^−/−^ mouse flash responses was investigated in 10 isolated retinas. The responses were recorded while perfusing the isolated retina with normal Ca^2+^ solution (containing 1 mM Ca^2+^) and then with low Ca^2+^ solution (containing ~ 20 nM free Ca^2+^). In 5 experiments, the low Ca^2+^ solution was changed back to normal Ca^2+^ solution and the flash responses were recorded again. Complete response families covering the whole operation range of rods were collected in each solution for all but one retina. Additionally, in 4 experiments, response families were collected only in low Ca^2+^ solution (giving a total number of low Ca^2+^ response families of 13). One to twenty-one responses were averaged with each flash strength depending on the response amplitude and noise level. The derived parameter values (see Table 1) were compared by using two-tailed paired student’s t-test. In all tests, p-value below 0.05 was considered statistically significant.

**Table 1:** Parameter values determined from responses recorded by LERG-OS first in normal Ca^2+^ and then in low Ca^2+^ from 10 experiments. In 5 experiments, responses were also recorded in normal Ca^2+^ after the washout of low Ca^2+^ solution.

Parameter/Treatment	*r*_*max*_ (µV)	*S*_F,*dark*_ (%/R*rod^−1^)	*Φ*_1/2_ (R*rod^−1^)	*τ*_*rec*_ (ms)	*t*_*p*_ (ms)	*t*_*integr.*_(ms)	τ_D_ (ms)	*A* (s^−2^)
Normal Ca^2+^ (n = 10)	45.9 ± 6.3, *P* = 0.006	4.7 ± 0.5, *P* = 0.003	15.6 ± 2.4, *P* = 0.0008 (n = 9)	348 ± 20, *P* = 0.017	270 ± 7, *P* = 0.0004	619 ± 35, *P* = 0.005	194 ± 6, *P* = 0.48	19.1 ± 2.3, *P* = 0.158
Low Ca^2+^ (n = 10)	79.3 ± 8.2	3.8 ± 0.5	18.7 ± 2.5 (n = 9)	287 ± 15	254 ± 7	538 ± 24	197 ± 6	20.3 ± 2.0
Normal Ca^2+^, wash (n = 5)	45.5 ± 3.7, *P* = 0.008	4.4 ± 0.6, *P* = 0.03	12.8 ± 2.0, *P* = 0.056 (n = 4)	355 ± 24, *P* = 0.033	284 ± 9, *P* = 0.022	670 ± 33, *P* = 0.068	196 ± 7, *P* = 0.51	19.2 ± 3.0, *P* = 0.159

*r*_max_, saturated response amplitude measured from the LERG-OS response plateau level; *S*_*F,dark*_, Fractional sensitivity, dim flash response amplitude divided with the strength of the flash stimulus and saturated response amplitude; *Φ*_1/2_, half-saturating flash strength; *τ*_rec_, time constant of single exponential fit to the recovery phase of dim flash responses; *t*_*p*_, time-to-peak of dim flash response; *t*_integr_, integration time of dim flash response calculated by taking the integral from time of the flash stimulus to the time when the response has completely recovered and dividing the value with the maximal amplitude of the dim flash response; τ_D_, the dominant time constant of saturated response recovery obtained from Pepperberg plot; *A*, amplification constant determined by fitting the flash response model to the response leading edges (Eq. (15)) of response families from individual experiments, as shown in Fig. 2. The lifetime of light-activated rhodopsin was locked to 28 ms in normal Ca^2+^ and to 26 ms in a low Ca^2+^ solution. The statistical significance was tested using two-tailed paired student’s t-test. The parameters derived in normal Ca^2+^ were first compared to those derived in low Ca^2+^ and secondly the parameters derived in normal Ca^2+^ after the washout were compared to low Ca^2+^ parameters derived in the experiments were the washout was performed. The parameter values are presented as mean ± SEM.

The inhibition constants against light-activated and basally activated PDE6, and the basal PDE6 activity with cGMP clamp were determined in normal Ca^2+^ (n = 9 retinas) and in low Ca^2+^ solution (n = 7 retinas) as described in^19^. The cGMP clamp experiments in normal and low Ca^2+^ were conducted with separate retinas. All experiments lasted less than 2.5 h. The statistical significance of the calcium-induced inhibition constant modulation against light-activated PDE6 by calcium was tested using a two-tailed unpaired t-test with unequal variances. Each retina was considered as one observation. The basal PDE6 activity and the inhibition constant against basally activated PDE6 were determined by pooling the data from each retina and calculating the mean value and the 95% confidence limits or the standard error of regression for the parameters.

### Theoretical background and calculations

The phototransduction models used in this study and the theoretical background of cGMP clamp have been described in detail in^19^, while the methods for determining the phosphodiesterase inhibitor inhibition constants are published in^26^. Here we give a summary of the utilized phototransduction models and the core ideas behind the methods.

#### cGMP clamp

In the rod photoreceptor cytoplasm without any stimulating light, the rate of cGMP synthesis by guanylate cyclase, $\alpha_{dark}$, and the rate constant of cGMP hydrolysis by basal PDE6 activity, $\beta_{dark}$, determine the steady-state intracellular cGMP concentration^8,27,28^.

1 $$\frac{{d \left[ {cGMP} \right]\left( t \right)}}{dt} = \alpha_{dark} - \beta_{dark} [cGMP]_{dark} = 0$$

The PDE6 activity can be decreased by introducing a phosphodiesterase inhibitor to the rod cytoplasm. A competitive PDE6 inhibitor decreases the catalytic activity of both basally activated PDE6 and light-activated PDE6 with inhibition constants $K_{I,dark}$ and $K_{I,light}$, respectively. The introduction of a PDE6 inhibitor in darkness decreases the cGMP hydrolysis by basally activated PDE6 and thereby leads to an elevation in the intracellular cGMP concentration. This elevation can be prevented by increasing PDE6 activity with light. By adding just the right amount of light, the cGMP concentration can be clamped to its value in darkness. Hence, in the presence of phosphodiesterase inhibitor and compensating light, we can derive

2 $$\frac{{d \left[ {cGMP} \right]\left( t \right)}}{dt} = \alpha_{dark} - \left( {\frac{{\beta_{dark} }}{{1 + \frac{\left[ I \right]}{{K_{I,dark} }}}} + \frac{{\beta_{light} }}{{1 + \frac{\left[ I \right]}{{K_{I,light} }}}}} \right)\left[ {cGMP} \right]_{dark} = 0,$$

where $1/\left( {1 + \frac{\left[ I \right]}{{K_{I,i} }}} \right)$ denotes the decrease of PDE6 activity due to the introduction of competitive PDE6 inhibitor,$\left[ I \right]$ is the inhibitor concentration, and $\beta_{light}$ stands for the increment in the PDE6 activity due to background light. Combining Eqs. (1) and (2) yields a formula that can be used to determine the value for $\beta_{dark}$.

3 $$\beta_{dark} = \beta_{light} /\left( {{\raise0.7ex\hbox{${1 + \frac{\left[ I \right]}{{K_{I,light} }}}$} \!\mathord{\left/ {\vphantom {{1 + \frac{\left[ I \right]}{{K_{I,light} }}} {1 + \frac{{K_{I,dark} }}{\left[ I \right]}}}}\right.\kern-\nulldelimiterspace} \!\lower0.7ex\hbox{${1 + \frac{{K_{I,dark} }}{\left[ I \right]}}$}}} \right)$$

$\beta_{light}$ can be calculated based on certain phototransduction parameter values and on the amount of light needed to clamp the cGMP to its dark-value, $\Phi_{BG}$ expressed as rhodopsin isomerizations per rod per second (R*rod^−1^ s^−1^)^19^:

4 $$\beta_{light} = \Phi_{BG} \frac{{A\tau_{R} \tau_{PDE} }}{{n_{cGMP} }},$$

where $\tau_{R}$ and $\tau_{PDE}$ are the average lifetimes of activated rhodopsin and PDE6, respectively, and $n_{cGMP}$ is Hill’s coefficient for CNG channels. $A$ is the rod amplification constant that describes the molecular amplification of phototransduction,

5 $$A = \nu_{RE} \beta_{sub} n_{cGMP} ,$$

where $\nu_{RE}$ denotes the rate constant by which the activation of rhodopsin leads to the activation of PDE6 subunits, and $\beta_{sub}$ is the catalytic efficacy of a single light-activated PDE6 subunit. Next, we will briefly show how the parameter values needed for the cGMP clamp can be derived.

### Determination of inhibition constants for phosphodiesterase-6 inhibitors

#### The inhibition constant against light-activated PDE6

PDE6 inhibitors reduce the rate by which the PDE6 enzyme hydrolyzes cGMP. When a competitive inhibitor of PDE6 is introduced into the photoreceptor cell, it will reduce the rate of light-induced cGMP hydrolysis according to an equation

6 $$\beta_{sub,I} = \frac{{\beta_{sub} }}{{1 + \frac{\left[ I \right]}{{K_{I,light} }}}},$$

where $\beta_{sub,I}$ is the rate constant for the catalytic activity of a single “light-activated” PDE6 subunit in the presence of inhibitor. The reduction of the light-induced PDE6 hydrolytic activity leads to a decrease in the molecular amplification of phototransduction

7 $$A_{I} = \nu_{RE} \beta_{sub,I} n_{cGMP} = \nu_{RE} \left( {\frac{{\beta_{sub} }}{{1 + \frac{\left[ I \right]}{{K_{I,light} }}}}} \right)n_{cGMP} .$$

The ratio of amplification constants without and in the presence of inhibitor yields a linear equation that can be used to determine the inhibition constant for PDE6 inhibitors against light-activated PDE6, $K_{I,light}$^26^:

8 $$\frac{{A_{control} }}{{A_{I} }} = \frac{\left[ I \right]}{{K_{I,light} }} + 1$$

#### The inhibition constant against basal PDE6 activity

Introduction of PDE6 inhibitor reduces the basal PDE6 activity from $\beta_{dark}$ to $\beta_{dark,I}$ and, subsequently, increases the intracellular cGMP concentration from $[cGMP]_{dark}$ to $\left[ {cGMP} \right]_{dark,I}$. In GCAPs^−/−^ mice, the guanylate cyclase-activating proteins have been knocked out, and the cGMP synthesis rate by guanylate cyclase, $\alpha_{dark}$, is constant. The following relation holds after reaching a steady-state in the absence and in the presence of phosphodiesterase inhibitor, respectively,

9 $$\frac{{d \left[ {cGMP} \right]\left( t \right)}}{dt} = \alpha_{dark} - \beta_{dark} [cGMP]_{dark} = \alpha_{dark} - \beta_{dark,I} \left[ {cGMP} \right]_{dark,I} = 0.$$

For a competitive PDE6 inhibitor, Eq. (9) gives

10 $$\frac{{\left[ {cGMP} \right]_{dark,I} }}{{\left[ {cGMP} \right]_{dark} }} = \frac{{\beta_{dark} }}{{\beta_{dark,I} }} = 1 + \frac{\left[ I \right]}{{K_{I,dark} }}.$$

The relative change in the circulating current in rods, and thus the extracellular voltage across the rod outer segment layer, is proportional to the relative change in the intracellular cGMP concentration to the power of the Hill’s coefficient for CNG channels (see, e.g.,^8^). Thus, $K_{I,dark}$ can be determined from the relation^26^_:_

11 $$\left( {\frac{{r_{max, I} }}{{r_{max,control} }}} \right)^{{{\raise0.7ex\hbox{$1$} \!\mathord{\left/ {\vphantom {1 {n_{cGMP} }}}\right.\kern-\nulldelimiterspace} \!\lower0.7ex\hbox{${n_{cGMP} }$}}}} = \frac{\left[ I \right]}{{K_{I,dark} }} + 1,$$

where $r_{max,I}$ and $r_{max, control}$ present the maximal light-inducible LERG-OS voltage change in the presence and in the absence of the phosphodiesterase inhibitor, respectively.

#### Modeling photoresponses

After a flash stimulus, both rhodopsin and PDE6 activation can be assumed to decay with first-order reaction kinetics^8^. For rhodopsin, this approximation is not mechanistically accurate since deactivation of rhodopsin is known to proceed through multiple phosphorylation steps by rhodopsin kinase and to cease to an arrestin binding to phosphorylated rhodopsin^29–38^. However, we presume that the approximation has only a small effect on the early activation phase of rod responses. The implications from the single exponential approximation and modeling of rhodopsin deactivation are discussed in^19,37,39^. The increment in the PDE6 activity caused by a flash of light, $\beta_{light}$, PDE6 can be calculated based on the convolution

12 $$\beta_{light} \left( t \right) = \beta_{sub} PDE6^{*} \left( t \right) = \beta_{sub} \Phi e^{{ - \frac{t}{{\tau_{R} }}}} *\nu_{RE} e^{{ - \frac{t}{{\tau_{PDE} }}}} .$$

$\Phi$ describes the flash stimulus strength as rhodopsin isomerizations per rod (R*rod^−1^)^8^. The change in the cGMP concentration after a flash stimulus can be described by the differential equation that takes into account the synthesis of cGMP by guanylate cyclase and the hydrolysis of cGMP by both basal and light-activated PDE6 activity,

13 $$\frac{{d\left[ {cGMP} \right]\left( t \right)}}{dt} = \alpha - \left( {\beta_{dark} + \beta_{light} \left( t \right)} \right)\left[ {cGMP} \right]\left( t \right).$$

Additionally, the LERG-OS flash response waveform *r*(*t*) depends on the relative change in the cytoplasmic cGMP concentration in the following way:

14 $$\frac{r\left( t \right)}{{r_{max} }} = 1 - \left( {\frac{{\left[ {cGMP} \right]\left( t \right)}}{{\left[ {cGMP} \right]_{dark} }}} \right)^{{n_{cGMP} }} ,$$

where $\frac{r\left( t \right)}{{r_{max} }}$ is the amplitude of the flash response normalized with the saturation amplitude. Now, model photoresponses to flash stimuli of light can be obtained by calculating $\beta_{light} \left( t \right)$ according to Eq. (12), solving $\left[ {cGMP} \right]\left( t \right)$ from the differential Eq. (13) numerically and transforming the calculated relative change in the cGMP concentration to a change in relative LERG-OS response waveform according to Eq. (14). These equations are valid if the rod outer segments are well-stirred, i.e., there are no significant concentration gradients, if there are no calcium-mediated feedback mechanisms present, and if the protein concentrations do not change significantly during the photoresponses. We assume that such conditions apply for dim flash responses of GCAPs^−/−^ recoverin^−/−^ mice^40^. Additionally, cell-to-cell variations are averaged out in the mass potential ERG signal, which reduces the deviation between experiments.

When modeling photoresponses, the values for several free parameters need to be estimated: The amplification constant of phototransduction (*A*), the lifetimes of activated rhodopsin $\left( {\tau_{R} } \right)$ and PDE6 $\left( {\tau_{PDE} } \right)$, the basal PDE6 activity $\left( {\beta_{dark} } \right)$, the cytoplasmic cGMP concentration in darkness $\left( {\left[ {cGMP} \right]_{dark} } \right)$, the guanylate cyclase activity (*α*), and the Hill’s coefficient for CNG channels ($n_{cGMP}$). In the literature, $n_{cGMP}$ is accepted to lie close to 3 in rod photoreceptors^8,41^. In GCAPs^−/−^ mouse rods, the guanylate cyclase is assumed to synthesize cGMP with a constant rate, the activity α being near 16.7 µMs^−1^ ^41^. These two values were fixed in our modeling. The cytoplasmic cGMP concentration in darkness can be obtained from the ratio of rate constants for cGMP synthesis and hydrolysis, $\left[ {cGMP} \right]_{dark} = \alpha /\beta_{dark}$. Thus, if a decrease in intracellular Ca^2+^ concentration were to increase $\beta_{dark}$, it would also cause a drop in $\left[ {cGMP} \right]_{dark}$. The dominant time constant of saturated response recovery $\left( {\tau_{D} } \right)$ describes the average lifetime of light-activated PDE6 in mouse rods^42–44^. $\tau_{D}$ can be determined using the Pepperberg plot analysis where the time intervals responses stay in saturation are plotted against the natural logarithm of flash strength^44^. The lifetime of activated rhodopsin is substantially shorter than the lifetime of activated PDE6 in mouse rods^42,45^. Based on suction electrode recordings, it is estimated that the lifetime of rhodopsin lies close to 40 ms in bicarbonate-buffered solution^45^ while our LERG-OS recordings conducted in HEPES-buffered solution gave an estimate close to 50 ms for wild type and GCAPs^−/−^ mice^19^. We also found that knocking out recoverin in GCAPs^−/−^ background decreased the rhodopsin lifetime to 28 ms^19^. Such a short $\tau_{R}$ makes it challenging to determine the amplification constant of mouse rods utilizing the Lamb and Pugh activation model^46^, because the rhodopsin deactivation starts to affect already the early response rising phase. Consequently, we can derive an optimal combination of *A* and $\tau_{R}$ by modeling the response rising phase though neither of the parameters can be determined fully independently. It is worth noting that with the high turnover rate of cGMP in mouse rods, set largely by the basal PDE6 activity, also the cGMP synthesis starts to affect the responses quite early on during the response onset. Still, for a brief moment after the stimulus, it can be approximated that the response onset is mainly determined by the phototransduction activation reactions together with the deactivation reactions of activated rhodopsin and PDE6 (included in the term $\beta_{light} \left( t \right)\left[ {cGMP} \right]\left( t \right)$ in Eq. (13)), while the hydrolysis of cGMP by basal PDE6 activity ($\beta_{dark} \left[ {cGMP} \right]\left( t \right)$ in Eq. (13)) and the synthesis of cGMP by guanylate cyclase ($\alpha = \beta_{dark} [cGMP]_{dark} ,$ constant in GCAPs^−/−^ rods) play only a minor role. This approximation holds when $\beta_{light} \left( t \right)\left[ {cGMP} \right]\left( t \right) \gg \alpha - \beta_{dark} \left[ {cGMP} \right]\left( t \right)= \beta_{dark} \left( {\left[ {cGMP} \right]_{dark} - \left[ {cGMP} \right]\left( t \right)} \right),$ and with this assumption, Eq. (13) simplifies to a form

15 $$\frac{{d\left[ {cGMP} \right]\left( t \right)}}{dt} \approx \beta_{light} \left( t \right)\left[ {cGMP} \right]\left( t \right).$$

Now, the response onset is described by four parameters: $A$, $\tau_{R}$, $\tau_{PDE}$, and $n_{cGMP}$. We analyzed earlier that the error in modeling photoresponse onset when using Eq. (15) instead of Eq. (13) is less than 10% within a range of 34 ms from the flash stimulus^19^. The analysis presumes that the modeled response is sub-saturated and that the basal PDE6 activity is lower than 6 s^−1^. Here, we use Eq. (15) with the $\tau_{PDE}$ determined by Pepperberg plot analysis and the literature value for $n_{cGMP}$ to determine the values of rhodopsin lifetime ($\tau_{R}$) and the amplification constant $A$. These parameter values are used to determine $\beta_{dark}$ (1) from the cGMP clamp paradigm, and (2) by modeling the whole fractional dim flash responses of DKO mouse retina, i.e., the dim flash responses divided by the LERG-OS saturation amplitude and flash stimulus strength.

## Results

### Low Ca^2+^ causes acceleration of flash response recovery in DKO mouse retinas

**Figure 1:**
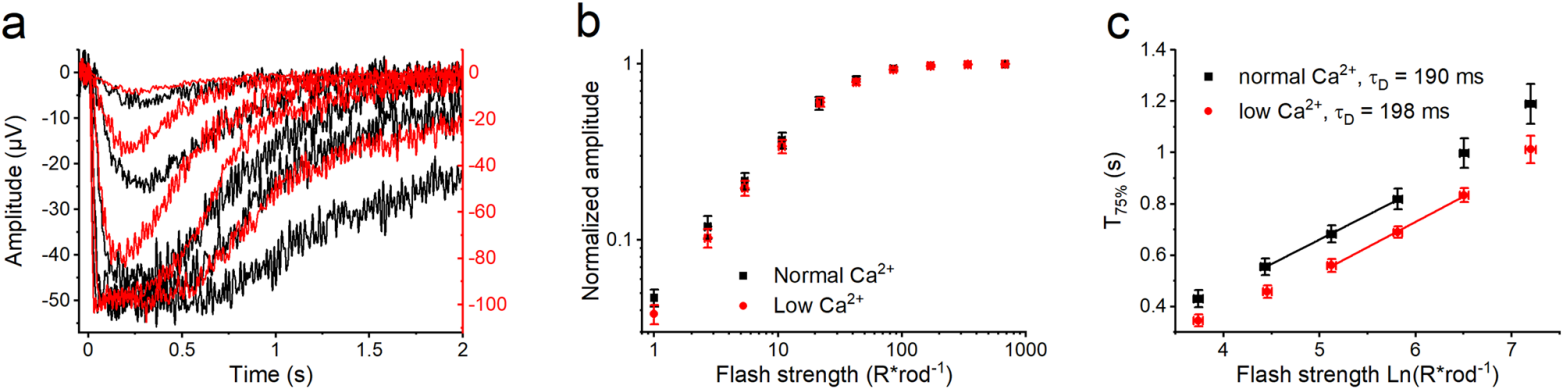
Effect of low Ca^2+^ on mouse rod flash responses recorded with LERG-OS. (**a**) Response families first recorded in normal Ca^2+^ (black traces and left y-axis) and then in low Ca^2+^ solution (red traces and right y-axis). The flash strengths were 2.64, 10.5, 41.9, 167, and 664 R*rod^−1^ in both solutions and responses are averages from 1 to 7 repetitions. (**b**) Operation curves for rod flash responses recorded in normal Ca^2+^ (black squares) and in low Ca^2+^ (red circles) solution normalized with the LERG-OS saturation amplitude. Each symbol presents mean value from 9 experiments. The first symbol from left illustrates a calculated average single-photon response (dim flash response divided with the stimulus strength). (**c**) Pepperberg plot where the time points of saturated response return to 75% level (T_75%_) are plotted against the natural logarithm of the flash strength. The dominant time constant of the saturated response return is determined as a linear fit to data. Symbols present mean values from 7 to 10 experiments. In both (**b**) and (**c**) responses were always recorded first in normal and then in low Ca^2+^ solutions. The error bars present SEMs.

In a previous study, we demonstrated that fast light adaptation in mouse rod photoreceptors is completely mediated by calcium ions^16^. Further, we showed that even after knocking out the calcium sensor proteins GCAPs and recoverin, calcium can still modulate light sensitivity and photoresponse kinetics in mouse rods, suggesting a remaining unknown mediator of rod light adaptation^16^. To illustrate the phenomenon, Fig. 1a shows LERG-OS flash responses recorded in darkness from a GCAPs^−/−^ recoverin^−/−^ double knockout mouse (DKO) retina, in normal Ca^2+^ conditions (1 mM, black traces) and after the extracellular calcium concentration was dropped to ~ 20 nM (red traces). The low extracellular Ca^2+^ is expected to drive the intracellular calcium in rods well below that during rod saturating light (near 20 nM^10^). Hence, the low Ca^2+^ treatment can be expected to drive the intracellular calcium sensor proteins out of their physiological operation range and to uncover any remaining component of calcium-mediated light adaptation, as found in^16^. Despite the absence of GCAPs and recoverin, the lowering of Ca^2+^ was still able to accelerate flash response recovery without affecting the activation phase (Fig. 1a). This resulted in a drop in fractional sensitivity and mild steepening of the rod operation curve (Fig. 1b and Table 1). In addition, low Ca^2+^ generated a substantial increase in the LERG-OS saturation amplitudes, implying an increase in the circulating dark current of rod outer segment ^22^. All these effects were reversed when the nutrition solution was returned from low Ca^2+^ to the solution with normal Ca^2+^ (Table 1).

### Lowering Ca^2+^ does not affect the lifetimes of activated PDE6 or rhodopsin in DKO mouse retinas

The lifetimes of activated rhodopsin and PDE6 are two key factors regulating the shut-off of flash responses. In mouse rods, the dominant time constant of saturated response recovery $\left( {\tau_{D} } \right)$ describes the average lifetime of activated PDE6 $\left( {\tau_{PDE} } \right)$ and can be determined from Pepperberg plot analysis^42–44^. In the DKO mouse retinas, the Pepperberg plots gave similar $\tau_{D}$ values in normal and low Ca^2+^ conditions (Fig. 1c and Table 1). This result is in line with the results from earlier studies suggesting that the modulation of $\tau_{D}$ requires the presence of recoverin in mouse rods^13,15,16^.

To examine whether the lifetime of activated rhodopsin might be modulated by reductions in the intracellular Ca^2+^ in DKO mouse retinas (i.e., in the absence of recoverin), we compared the early phase of flash responses recorded in normal and in low Ca^2+^ conditions by fitting the flash response model to the first 34 ms of the response leading edge (Eq. (15)). This range for model validity has been earlier determined in^19^. Within this range, the response onset is mainly determined by the phototransduction activation reactions as well as the deactivation rates of light-activated rhodopsin and PDE6 while the hydrolysis of cGMP by basal PDE6 activity and synthesis of cGMP by guanylate cyclase compensate for each other and can be disregarded.

**Figure 2:**
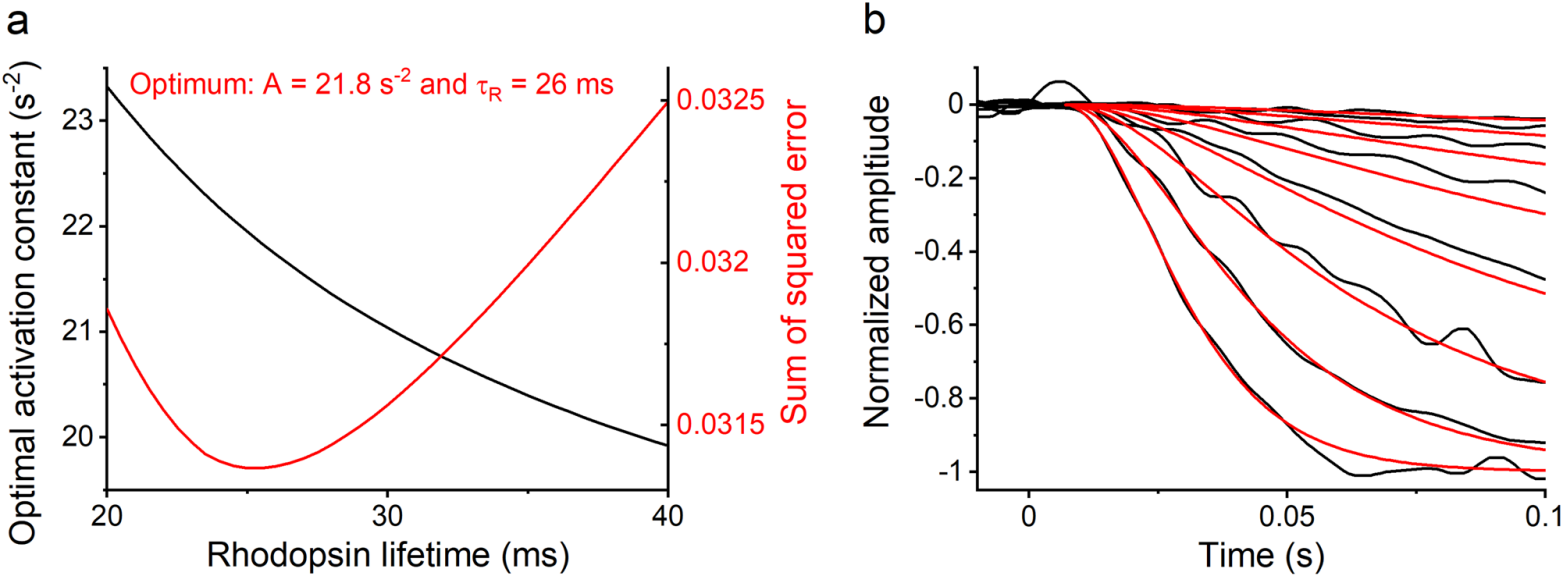
Determination of rhodopsin lifetime and phototransduction amplification constant by fitting the flash response model to the response leading edge (Eq. (15)) to the activation phase of the population-averaged LERG-OS response family recorded in low Ca^2+^ solution with twofold increments in flash strengths. (**a**) The outcome of fitting the model with different τ_R_ values to the response family. The black curve shows the optimal amplification constants leading to the best fit with different τ_R_ values, and the red curve shows the sum of squared errors of the fits. The combination of τ_R_ and *A* values leading to the overall best fit (lowest sum of squared error, the minimum of the red curve) of the model was τ_R_ = 26 ms and *A* = 21.8 s^−2^. (**b**) *A* population-averaged response family in low Ca^2+^ (n = 13) and the model fittings using τ_R_ = 26 ms and *A* = 21.8 s^−2^. Response families were recorded with flash strengths ranging from 1.4 to 177 R*rod^−1^. The model was fitted to all responses up to the first saturated response.

Figure 2 shows the results of fitting the flash response model to the response leading edge (Eq. (15)) of the population-averaged flash response family from all the 13 retinas recorded in low Ca^2+^ conditions. The $\tau_{D}$ of 198 ms determined from this response family was used as the lifetime of PDE6 in the modeling. The model was fitted by sweeping the rhodopsin lifetime $\tau_{R}$ through fixed values between 20 and 40 ms with 0.5 ms interval and determining the optimal amplification constant $A$ with each $\tau_{R}$. The combination of $\tau_{R}$ and $A$ that gave the lowest sum-squared error of the fit was chosen for later analysis. Figure 2a shows the relation between the rhodopsin lifetime $\tau_{R}$ and the amplification constant $A$ in the fittings as well as the evolution of the sum-squared error of the fits. We obtained the best fit with the rhodopsin lifetime of 26 ms and the amplification constant of 21.8 s^−2^. Figure 2b shows the fits to the early phase of flash responses with the optimal $\tau_{R}$ and $A$ values. A similar analysis was conducted for responses recorded in normal Ca^2+^ in^19^, where the optimal fit to population-averaged response families was achieved with rhodopsin and PDE6 lifetimes of 28 ms and 196 ms, respectively, and with an amplification constant of 18.9 s^−2^ (see Fig. 5e and f in^19^). The comparison of these analyses suggests that neither rhodopsin lifetime, PDE6 lifetime, nor phototransduction amplification constant change significantly in DKO mouse rods due to the reduction in intracellular Ca^2+^ concentration.

To analyze the key parameters of the dataset in normal and low Ca^2+^, rhodopsin lifetime was fixed to the value determined by modeling, $\tau_{D}$ was determined from the Pepperberg plot for each experiment, and the amplification constant was defined by fitting the flash response model to the response leading edges (Eq. (15)) separately for each retina. Table 1 shows average parameter values from those 10 DKO retinas that were examined both in normal Ca^2+^ and in low Ca^2+^ solution. For 5 out of the 10 retinas, control recordings were conducted in normal Ca^2+^ solution also after the low Ca^2+^ treatment. We observed no substantial differences between the parameter values obtained before the introduction and after the washout of low Ca^2+^ solution.

### Effect of PDE6 inhibitor on intracellular cGMP level can be annulled with light using cGMP clamp paradigm

Since the acceleration of photoresponse shut-off in low Ca^2+^ shown in Fig. 1 is not caused by the shortening of the lifetimes of rhodopsin or PDE6, the remaining candidate is the increased turnover rate of cGMP either by dynamic elevation in guanylate cyclase activity or increased basal PDE6 activity^8,41,47^. In DKO mouse retinas, the proteins regulating guanylate cyclase activity, GCAPs, are knocked out, and therefore, no calcium-mediated modulation of guanylate cyclase activity should remain. Hence, a potential source for the low Ca^2+^ induced acceleration of flash response kinetics might be the increased basal PDE6 activity. To test this hypothesis, we conducted cGMP clamp experiments with DKO mouse retinas in normal and in low Ca^2+^ conditions.

**Figure 3:**
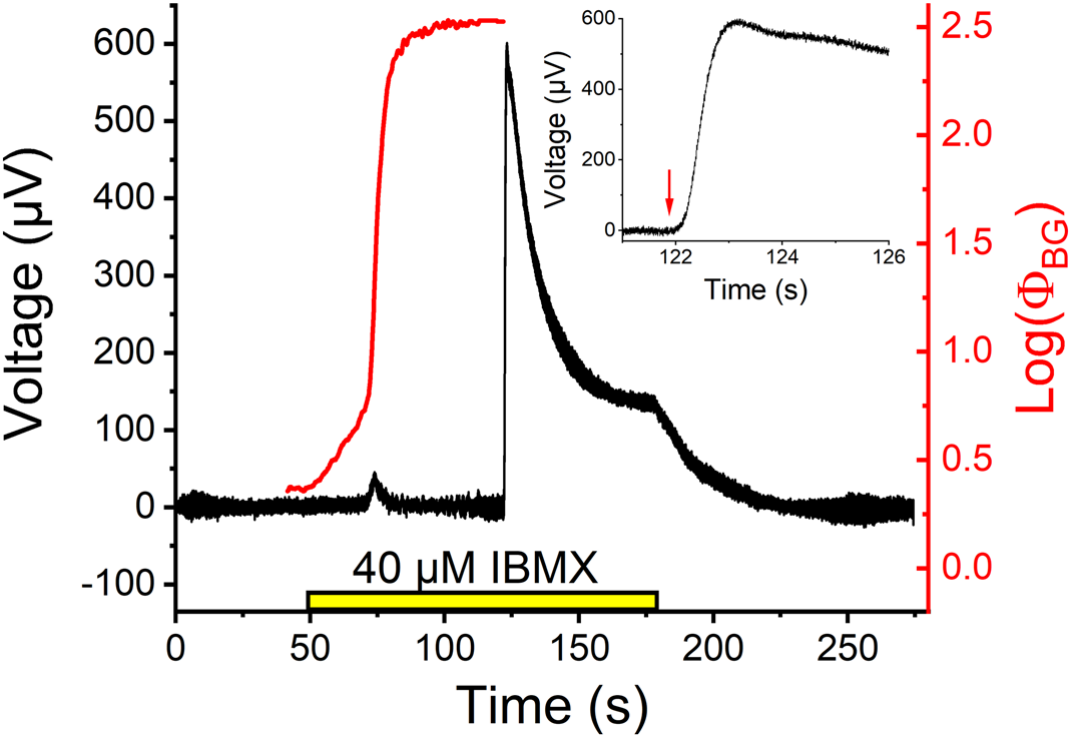
One cGMP clamp run recorded in normal Ca^2+^ with LERG-OS. The black trace illustrates the LERG-OS voltage and red trace the background light strength produced by the closed-loop PID-controlled feedback system to keep the LERG-OS voltage steady after the introduction of 40 µM IBMX (yellow bar). After the controller has reached a steady-state, the background light is turned off. This causes a rapid increase in the LERG-OS voltage, which downregulates to a new steady-state after maximal effect of IBMX (the peak voltage increase). After the washout of IBMX, the LERG-OS voltage returns to the baseline level. The inset shows the LERG-OS voltage on an extended time scale around the time of the turn-off of the background light. The red arrow indicates the precise time of the background turn-off.

The cGMP clamp experiments were conducted by recording the LERG-OS signal while illuminating the retina with closed-loop PID-controlled background light, which received its feedback from the recorded LERG-OS signal. One example of cGMP clamp runs is shown in Fig. 3. First, IBMX was introduced to the retina along with the nutrition solution (yellow bar). The PID controller kept the signal steady at the baseline level by modulating the intensity of the background light. After the PID-controlled background light (red curve) had reached a steady-state, the light was turned off, and the signal was let to increase freely in order to estimate the maximal increase in the circulating current (reflected by the maximal change in the LERG-OS signal) with the used IBMX concentration. Finally, IBMX was washed out.

### PDE6 inhibition constants of IBMX differ in normal and in low Ca^2+^

**Figure 4:**
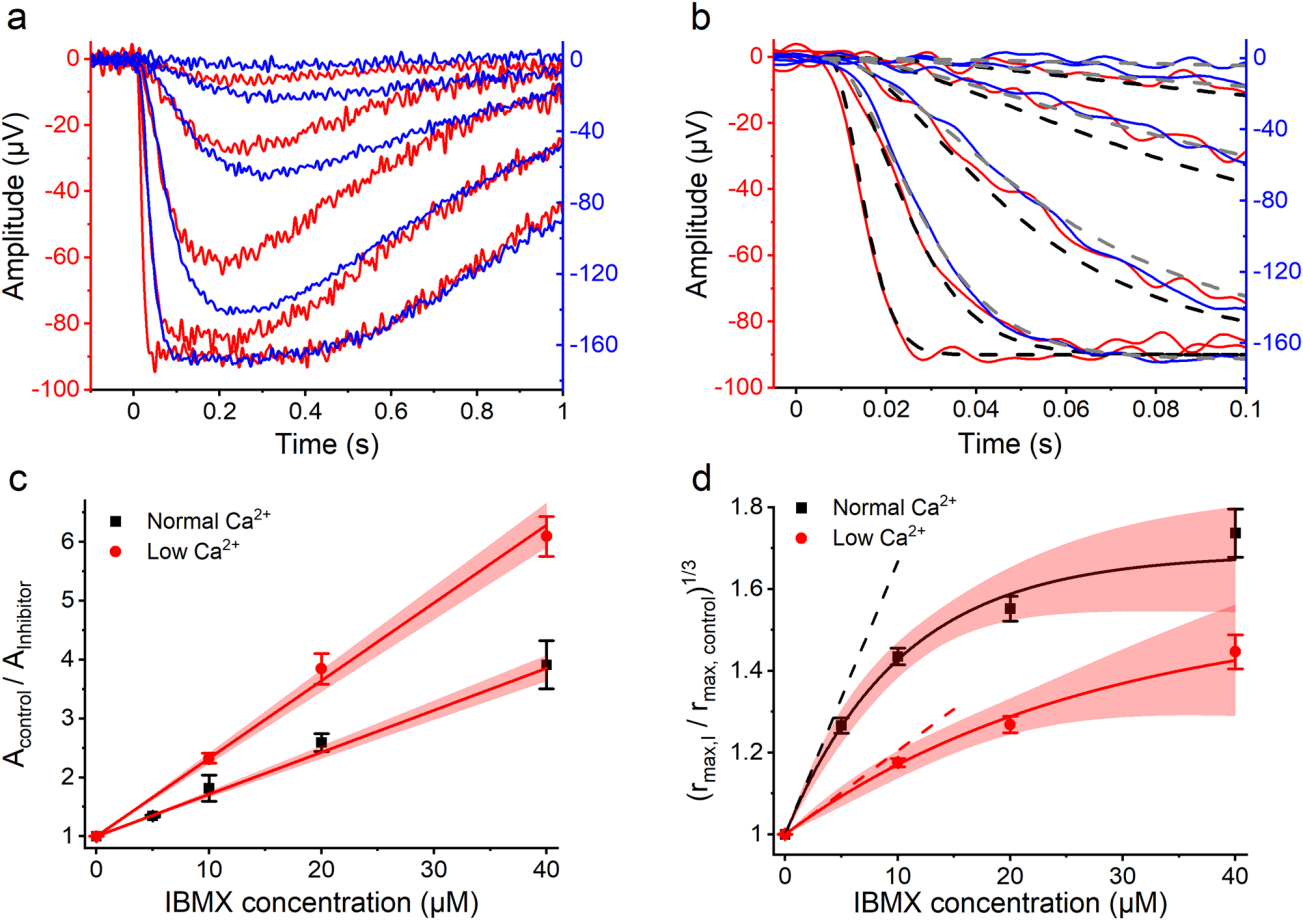
(**a**) Response families recorded in low Ca^2+^ solution without IBMX (red traces) and with 40 µM IBMX (blue traces). The flash strengths were 3.42, 13.6, 54.1, 216, and 858 R*rod^−1^ in both solutions and responses are averages from 1 to 3 repetitions. (**b**) Determination of amplification constants for response families shown in Fig. 4a by fitting the flash response model to the response leading edge (Eq. (15)) to the activation phase of the responses. *τ*_*R*_ was locked to 26 ms and *τ*_*PDE*_ to 199 ms, which was determined separately for the concerned retina from Pepperberg plot. Constant delay of 7 ms was used for the fits. Fitting gave an amplification constant of 27.7 s^−2^ in low Ca^2+^ without IBMX (black dashed lines) and 4.9 s^−2^ in 40 µM IBMX (gray dashed lines). (**c**) Determination of inhibition constant against light-activated PDE6 (*K*_*I*,*light*_). Fitting of Eq. (8) to the pooled data gave an inhibition constant of 13.9 µM and 7.6 µM for normal and low Ca^2+^, respectively. The intercept of the linear fit is fixed to 1. (**d**) Determination of inhibition constant against basally activated PDE6 (*K*_*I*,*dark*_). The figure shows the relative increase in the maximal LERG-OS amplitude right after the light turn-off in the cGMP clamp (see Fig. 3). The exponential fits to the cGMP clamp data extrapolated to zero inhibitor concentration gave 15.0 µM and 49.0 µM for *K*_*I*,*dark*_ in normal and low Ca^2+^, respectively (see Eq. (11)).

In order to determine the basal PDE6 activity using the cGMP clamp paradigm, we needed to know the potency of IBMX in inhibiting PDE6 in its light-activated and basally active form both in normal and in low Ca^2+^ conditions. Figure 4 illustrates the determination of the inhibition constants for IBMX against light-activated PDE6 and basal PDE6 activity, as described earlier^19,26^. The inhibition constant against light-activated PDE6, $K_{I,light}$, was determined from the decrements in the amplification constant caused by IBMX at different concentrations (Eq. (8)). Figure 4a illustrates response families recorded in low Ca^2+^ without IBMX (red traces) and with 40 µM IBMX (blue traces). Figure 4b demonstrates fitting of Eq. (15) to the leading edges of the responses in Fig. 4a in order to determine the corresponding amplification constant values. Figure 4c illustrates the determination of $K_{I,light}$ from population-averaged data in normal Ca^2+^ (black squares, redrawn from^19^) and in low Ca^2+^ (red circles). The amplification constant decreases in both solutions as predicted by Eq. (8). Surprisingly, the inhibition constant against the light-activated PDE6 was 13.8 ± 1.7 µM (n = 9) in normal Ca^2+^ but only 7.6 ± 0.5 µM in low Ca^2+^ (n = 7). This difference was statistically significant (t(9) = 3.52, *P* = 0.007, two-tailed unpaired t-test with unequal variances).

The inhibition constant against the basal PDE6 activity was determined from the growth of the saturation amplitude caused by the IBMX-induced decrease in the basal PDE6 activity. A reduction in the basal PDE6 activity leads to an increase in the intracellular cGMP concentration (Eq. (11)) and in the circulating dark current, thereby causing an elevation of the LERG-OS saturation amplitude, *r*_*max*_. The LERG-OS saturation amplitude before the addition of the inhibitor, *r*_*max,control*_, was determined from the plateau level of LERG-OS response to a strong stimulus before the cGMP clamp procedure. The growth of the saturation amplitude was determined from the maximal increase in the LERG-OS voltage after the turn-off of the background light in the cGMP clamp (the peak value of the voltage increase in Fig. 3) and *r*_*max,I*_ was obtained by summing this growth to *r*_*max,control*_. The termination of the cGMP clamp lets the cGMP concentration increase towards a new equilibrium with speed determined by the rate of deactivation of light-activated PDE6 and the pace of cGMP production by guanylate cyclase. This approach is justified with GCAPs^−/−^ mice, where the Ca^2+^-mediated feedback to guanylate cyclase activity is removed, and the guanylate cyclase is running at a constant speed even though the outer segment current increases. However, rods cannot maintain such a high current and, especially with high IBMX concentrations, the increase in LERG-OS voltage falls short from the theoretical expectation. This is seen as downregulation of the LERG-OS amplitude after the initial peak following the termination of the cGMP clamp (Fig. 3).

Figure 4b presents the $K_{I,dark}$ determination from the population-averaged data in normal Ca^2+^ (black squares, redrawn from^19^) and in low Ca^2+^ (red dots). The downregulation of cGMP increase induced by high IBMX concentrations can be seen as the nonlinear behavior in Fig. 4b contrary to that predicted by the Eq. (11). We estimated the $K_{I,dark}$ by fitting an exponential decay model to the pooled data from all experiments and by extrapolating the slope of the fit to zero inhibitor concentration to minimize the effect of cGMP increase downregulation to $K_{I,dark}$ determination. The $K_{I,dark}$ values obtained were 15.0 µM ([11.4; 18.6] 95% confidence limits, n = 9) in normal Ca^2+^ and 49.0 µM ([31.8; 66.1] 95% confidence limits, n = 7) in low Ca^2+^. As described earlier, the $K_{I,light}$ and $K_{I,dark}$ values were not significantly different in normal Ca^2+^ solution ($K_{I,light}$ = 13.8 µM vs. $K_{I,dark}$ = 15.0 µM)^19^. However, in low Ca^2+^ conditions, the inhibition constants against light-activated and basal PDE6 activity were remarkably different ($K_{I,light}$ = 7.6 µM vs. $K_{I,dark}$ = 49 µM), the $K_{I,dark}$ being 6.5 times the $K_{I,light}$. In further analysis, the $K_{I,light}$ values were determined separately for each retina in normal and low Ca^2+^ conditions. The $K_{I,light}$ determined in normal Ca^2+^ was used also as the $K_{I,dark}$ in normal Ca^2+^. However, in low Ca^2+^, the $K_{I,dark}$ was always assumed to be 6.5 times larger than the $K_{I,light}$.

### Low Ca^2+^ induces an increase in basal PDE6 activity

**Figure 5:**
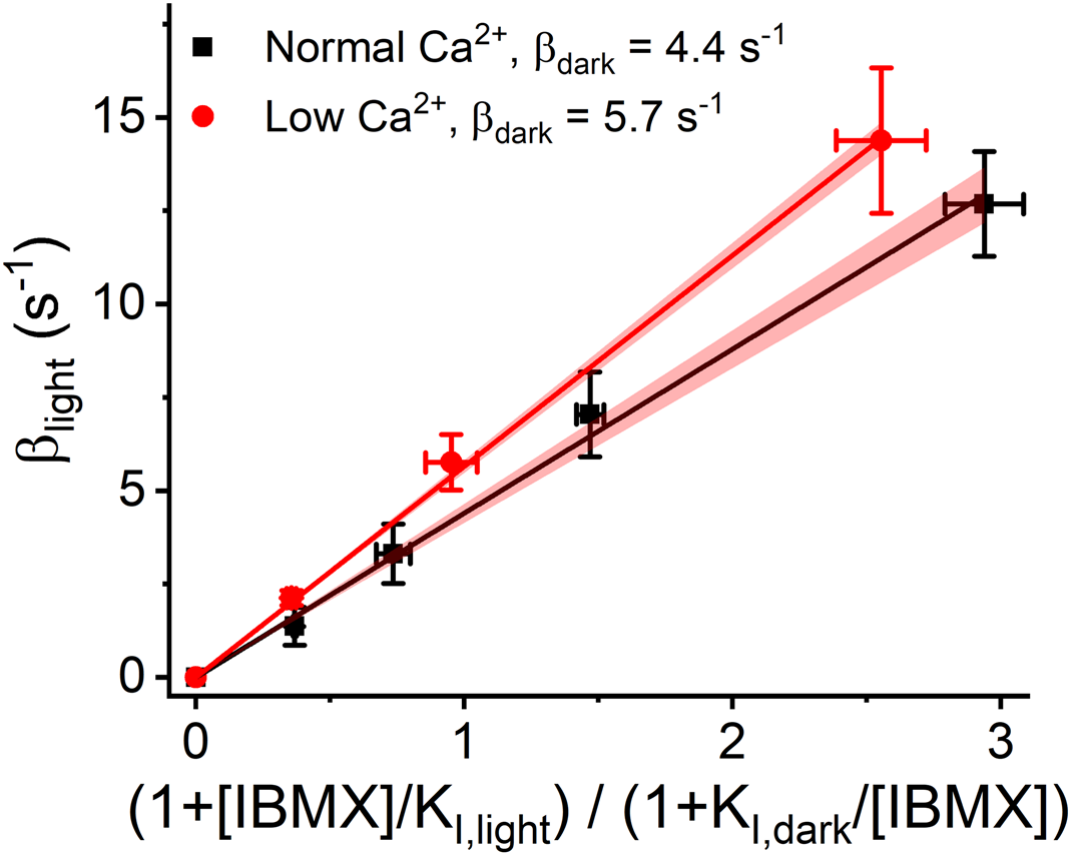
Determination of *β*_*dark*_ from cGMP clamp data. The linear fits to the data according to Eq. (3) gave *β*_*dark*_ values of 4.4 ± 0.1 s^-1^ (n = 9) and 5.7 ± 0.1 s^−1^ (n = 7, mean ± standard error of regression) for DKO mouse in normal and low Ca^2+^, respectively. The error bars present SEMs, and the shaded red area presents the 95% confidence intervals of the linear fits. The linear fit was forced to pass through the origin.

cGMP clamp experiments were conducted on 9 retinas in normal Ca^2+^ solution and on 7 retinas in low Ca^2+^ solution. Since the effects of IBMX were reversible at the concentrations used, we were able to conduct several cGMP clamp runs using 1–3 different IBMX concentrations with each retina. $\beta_{dark}$ was determined from the linear fit to the cGMP clamp data based on the Eq. (3). $\beta_{light}$ was calculated utilizing the knowledge of the strength of the background light needed in the cGMP clamp to reach the steady-state and the other determined parameter values shown in Eq. (4). The Hill coefficient for CNG channels, $n_{{{\text{cGMP}}}}$, was assumed to be 3 for mouse rods^8,41^. Figure 5 illustrates population-averaged cGMP clamp data with linear fits, along with the 95% confidence limits of the fits, to visualize the determination of the $\beta_{dark}$ in normal and in low Ca^2+^ conditions. The linear fits gave $\beta_{dark}$ values of 4.4 ± 0.1 s^−1^ in normal Ca^2+^ as previously reported in^19^ (mean ± SER, [4.1; 4.7] 95% confidence limits, n = 9) and 5.7 ± 0.1 s^−1^ in low Ca^2+^ solution (mean ± SER, [5.5; 5.8] 95% confidence limits, n = 7).

### Increase in basal PDE6 activity can explain the low Ca^2+^ induced rod desensitization

**Figure 6:**
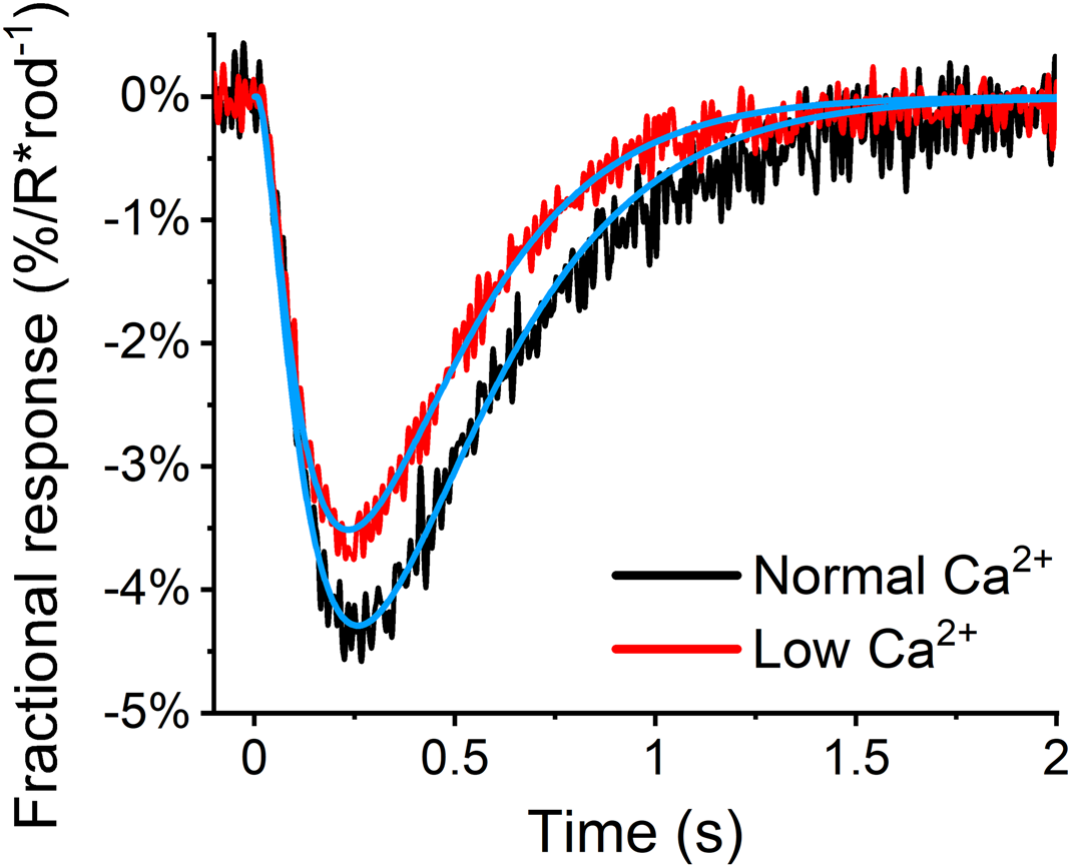
Determination of *β*_*dark*_ by model fitting (described in the Material and Methods, Eqs. (12) – (14)) to GCAPs^−/−^ recoverin^−/−^ mouse dim flash responses recorded with LERG-OS in normal and in low Ca^2+^. The dim flash responses are normalized by the saturation amplitude and the stimulus strength to obtain fractional responses. The dim flash responses are population averaged from 10 experiments. Responses were always recorded first in normal and then in low Ca^2+^ solution. The model fits are shown in light blue, and Table 2 shows the parameter values used in modeling.

To investigate whether the increase in $\beta_{dark}$ could explain the low Ca^2+^ induced desensitization of responses and acceleration of response shut-off kinetics, we modeled dim flash responses with a phototransduction model (Materials and Methods, Eq. (13)). Figure 6 shows population-averaged fractional dim flash responses from 10 retinas and the results of the modeling. In each retina, the responses were first collected in normal Ca^2+^ and then in the low Ca^2+^ solution. The rhodopsin and PDE6 lifetimes were locked to the values determined earlier. The Hill coefficient for CNG channels, $n_{{{\text{cGMP}}}}$, was assumed to be 3. The amplification constant and the $\beta_{dark}$ could vary freely. With the model premises, the guanylate cyclase activity and the cGMP concentration in darkness do not affect the relative change in response amplitudes in the modeling, and their value can be chosen freely as long as $\alpha = \beta_{dark} [cGMP]_{dark}$ applies. The best fit to the population-averaged fractional dim flash response in normal Ca^2+^ was achieved with $\beta_{dark}$ of 3.9 s^−1^ and in low Ca^2+^ with $\beta_{dark}$ of 5.0 s^−1^. Modeling fractional dim flash responses suggests that a 28% increase in $\beta_{dark}$ can explain the acceleration of response shut-off. This increase in $\beta_{dark}$ is quantitatively well in line with the cGMP clamp results giving an increase of 29% in $\beta_{dark}$ when introducing the low Ca^2+^ concentration. The parameter values used in the modeling are given in Table 2.

**Table 2:** The parameters used for fitting the phototransduction model in Fig. 6. The model was fitted to the responses from the moment of the flash stimulus to 600 ms after the stimulus.

Parameter		Units	Normal Ca^2+^	Low Ca^2+^
Lifetime of light-activated rhodopsin	$\tau_{R}$	ms	28	26
Lifetime of light-activated PDE6	$\tau_{PDE}$	ms	194	197
Amplification constant	*A*	s^−2^	19.5	19.0
Basal PDE6 activity	$\beta_{dark}$	s^−1^	3.9	5.0
The Hill coefficient for CNG channels	$n_{cGMP}$	–	3	3
Constant time delay	$t_{delay}$	ms	7	7
Fit length	–	ms	600	600

The cGMP level in darkness was calculated by dividing the synthesis rate of cGMP with the basal PDE6 activity. The mean τ_D_ values from Pepperberg plots in normal and low Ca^2+^ was used as the lifetime of light-activated PDE6. The amplification constant and the basal PDE6 activity could vary freely. The lifetime of light-activated rhodopsin was fixed to the values determined earlier.

## Discussion

In this study, we investigated the role of PDE6 in Ca^2+^-dependent regulation of phototransduction in mammalian rods. Our results support two major conclusions: (1) The basal PDE6 activity is not constant as previously thought but depends on calcium concentration. Low levels of calcium, mimicking the behavior of intracellular calcium in strong lights, increase the basal PDE6 activity. (2) Calcium modulates the PDE6 inhibition constant of the non-specific PDE inhibitor IBMX, revealing an interaction between Ca^2+^ and PDE6.

### Modulation of basal PDE activity

In a previous study, we demonstrated that fast light adaptation in mouse rods is completely mediated by calcium ions and that at least one unknown negative feedback mechanism must be involved in mouse rod phototransduction^16^. The phototransduction machinery of mammalian rods has two well-known negative feedback mechanisms mediated by the calcium sensor proteins GCAPs and recoverin^11–13^. Here we show that calcium can modulate rod flash responses even in GCAPs^−/−^ recoverin^−/−^ background. In our experimental approach, we lowered the extracellular free Ca^2+^ to around 20 nM. The Na^+^/Ca^2+^K^+^ exchange mechanism in the outer segment plasma membrane drives the intracellular Ca^2+^ concentration down towards the equilibrium value $\left[ {Ca^{2 + } } \right]_{in} = \frac{{[Na^{ + } ]_{in}^{4} \left[ {K^{ + } } \right]_{out} }}{{[Na^{ + } ]_{out}^{4} \left[ {K^{ + } } \right]_{in} }}\left[ {Ca^{2 + } } \right]_{out}$, presumably well below the level attainable by light in physiological conditions. Under the low Ca^2+^ conditions, the recovery of GCAPs^−/−^ recoverin^−/−^ mouse flash responses accelerated, leading to a decrease both in the fractional amplitudes and integration times of dim flash responses.

The acceleration of response recovery by low Ca^2+^ might be caused by modulation of several mechanisms: shortening of the lifetime of light-activated rhodopsin or PDE6, dynamic enhancement of guanylate cyclase activity, or increase in the basal PDE6 activity^8^. Since we made our investigations on the GCAPs^−/−^ recoverin^−/−^ mice, the acceleration of response deactivation cannot be mediated by calcium-dependent modulation of guanylate cyclase by GCAPs^11^, lifetime of rhodopsin^12–14^, or PDE6^13,15,16^ by recoverin. Additionally, switching between normal and low Ca^2+^ did not affect the dominant time constant of saturated flash response recovery, reflecting the lifetime of light-activated PDE6^42–44^. As Ca^2+^ did not modulate the dominant time constant, $$\tau_{D}$$ is consider to reflect the lifetime of light-activated PDE6 during both saturated and dim flash responses. The low Ca^2+^-induced acceleration of flash response kinetics was apparent in the recovery phase, while the early phase remained unchanged. Hence, we conclude that low Ca^2+^ did not affect phototransduction amplification. A third known calcium sensor protein, calmodulin, modulates the affinity of cGMP to CNG-channels in rods^17^. The $$K_{\raise.5ex\hbox{$\scriptstyle 1$}\kern-.1em/ \kern-.15em\lower.25ex\hbox{$\scriptstyle 2$} }$$ for calmodulin binding in rods is near 50 nM, and its Hill coefficient near 1.5^49,50^. Lowering the extracellular Ca^2+^ to ~ 20 nM should decrease the intracellular Ca^2+^ well below this value and drive calmodulin modulation out of its functional range, and thus, dynamic calmodulin-mediated cGMP affinity modulation during flash responses is unlikely in low Ca^2+^ solution. The low Ca^2+^-induced steady-state change in the affinity of cGMP to CNG-channels should manifest as modulation of absolute response amplitudes but would affect neither flash response amplitudes normalized with the saturation amplitude (as in Fig. 6) nor flash response kinetics^51^. Thus, none of the known three calcium sensor proteins involved in phototransduction can cause the discovered low Ca^2+^-induced acceleration of flash response recovery and decrease of the fractional amplitudes.

In the present study, we discovered, using the cGMP clamp procedure, that the basal PDE6 activity elevated by 29% when Ca^2+^ concentration was decreased. Based on this inference, we modeled the effect of elevated basal PDE6 activity on rod responses. Our modeling of dim flash responses showed that a 28% increase in basal PDE6 activity could explain the acceleration of photoresponse kinetics and the decrease in the fractional dim flash photoresponse amplitudes (see Fig. 6). This suggests that the low Ca^2+^-induced acceleration of flash response recovery is solely caused by increased basal PDE6 activity.

The observed ~ 30% increase in $\beta_{dark}$ should be accompanied by a corresponding decrease in the cGMP level in GCAPs^−/−^ retinas, which is expected to cause a two-fold decrease in the circulating dark current and LERG-OS amplitudes. Instead, we saw over 70% increase in the LERG-OS saturation amplitude when changed from normal to low Ca^2+^ conditions. This should, however, not contradict our interpretation: In the absence of GCAPs, the guanylate cyclase activity does not increase, and therefore the rise in $r_{max}$ by low Ca^2+^ most likely comes from the increase in the cation conductance of the outer segment plasma membrane. Elevation of the cation conductance, whether from the possible effect of low Ca^2+^ on the external Ca^2+^ binding sites in the CNG channel^52,53^ or from the calmodulin-mediated modulation of cGMP affinity to the CNG channel^17,54,55^, can neither explain the acceleration of the flash response kinetics nor affect the $\beta_{dark}$ determined by cGMP clamp. Hence, we believe that the low Ca^2+^-induced elevation of $\beta_{dark}$ and increase in LERG-OS amplitudes are two distinct phenomena.

The rod outer segment current seems to be independent of the membrane potential in the physiological voltage range in salamander rods^56^. However, a subtle voltage-dependence was discovered in pig rods^57^ and in mouse cones^58^. The low Ca^2+^ -induced increase in the CNG channel current depolarizes the cell membrane, which could influence the circulating current and LERG-OS responses. However, such an effect would likely be visible both in the response onset and recovery, while in our experiments, the low Ca^2+^ treatment affected only the response recovery. Additionally, in cGMP clamp experiments, the circulating current and membrane potential were held constant, and thus, membrane depolarization would not affect the cGMP clamp results. Since both response modeling and cGMP clamp gave coherent results, we consider plausible that neither membrane depolarization nor possible nonlinear current–voltage relation of CNG channels could significantly affect our experiments. Further, the low Ca^2+^ treatment can have multiple indirect effects on, e.g., cell metabolism, which can deteriorate the health of the retina. However, instead of deceleration of response kinetics, which is a typical indicator for deterioration of retinal health, the flash response kinetics accelerated in low Ca^2+^.

### Physiological role of basal PDE6 activity modulation by calcium

Dark-adapted rod photoreceptor cells are extremely sensitive to light and can detect absorptions of single photons. It has long been understood that the level of background noise sets the absolute threshold of vision and that molecular mechanisms in photoreceptor cells generate the majority of the “dark noise” in the retinal neural network^59,60^. The noise in photoreceptor cells consists of two components, discrete and continuous. The discrete noise is generated by spontaneous activations of the visual pigment molecules, producing signal waveforms indiscernible from responses to single photons. The continuous noise is believed to originate in fluctuations of the basal PDE6 activity due to thermal activations of PDE6 molecules^60^. Because the responses to spontaneous visual pigment activations cannot be distinguished from single-photon responses, the only way to prevent excessive rejection of real single-photon responses is to suppress discrete noise by making the visual pigment molecules very stable. In fact, the rhodopsin molecules in rod photoreceptor cells are believed to be among the most stable proteins in vertebrates, each rhodopsin experiencing a thermal activation once in several hundred years^40,61, 62^. However, a similar approach on continuous noise would lead to an suboptimal signal-to-noise ratio in photoreceptor cells. Decreasing the number of activated PDE6 subunits to less than one per rod compartment would cause occasional high fluctuations in cGMP concentration near that compartment when PDE6 is sporadically spontaneously activated. Additionally, lowering the basal PDE6 activity decreases the turnover rate of cGMP, and a very low cGMP turnover rate would lead to sluggish flash response kinetics and reduced temporal resolution of the visual system. On the other hand, increasing $\beta_{dark}$ also increases the needed number of light-activated PDE6 molecules to create a detectable signal, thus, reducing the absolute sensitivity of rods. Therefore, a moderate $\beta_{dark}$ is required to set the operation point of phototransduction. An optimal $\beta_{dark}$ is considered to be close to one active PDE6 subunit per compartment at given time^7,63^. The novel Ca^2+^-mediated modulation of $\beta_{dark}$ found in this study could allow tuning of $\beta_{dark}$ to an optimal value in order to optimize the rod signal-to-noise ratio near the absolute threshold of vision. In addition, Ca^2+^-mediated feedback on $\beta_{dark}$ can provide a mechanism to control the fluctuations in the cGMP concentration in the photoreceptor outer segment: When the $\beta_{dark}$ coincidentally decreases, it leads to an elevation in the intracellular cGMP concentration, which opens more CNG channels and increases the inward outer segment current that carries Na^+^ and Ca^2+^ ions into the cell. This raises the intracellular Ca^2+^ concentration and increase binding of Ca^2+^ ions to GCAPs causing reduction in cGMP synthesis by guanylate cyclase. Decreased intracellular cGMP concentration leads to a decrement in CNG channel current and intracellular Ca^2+^, which increases $\beta_{dark}$. On the other hand, when the $\beta_{dark}$ fluctuates towards higher values, the intracellular Ca^2+^ concentration decreases, leading to an increase in guanylate cyclase activity and to a decrease in $\beta_{dark}$. We suggest that these two mechanisms together can effectively dampen the fluctuations in the circulating current caused by spontaneous changes in basal PDE6 activity.

We interpret that the discovered 30% increase is the upper limit of the *β*_*dark*_ modulation attainable by calcium changes in mouse rods. These changes could increase the temporal resolution of rod vision in a bright background and partly explain the recent observations that mouse rods can remain functional in higher intensities than earlier appreciated^64–66^. However, the effect seems to be relatively weak and might not have a practical impact on the light adaptation of wild type rods with functional GCAPs- and recoverin-mediated light adaptation pathways. A more likely option is that the *β*_*dark*_ modulation is not essential in sustaining the rod sensitivity in background light but rather used to increase and stabilize the absolute rod sensitivity in darkness. Further, calcium-mediated GCAPs and recoverin independent light-adaptation has also been discovered from mouse cone photoreceptors^67^, and it remains yet to be investigated whether similar or stronger modulation of basal PDE6 activity is present in cones as we found in rods.

### Modulation of PDE6 inhibition by calcium

Cyclic nucleotide phosphodiesterases have a central role in a multitude of cellular signaling mechanisms, and therefore, specific inhibition of different PDE isoforms would have a great functional impact and therapeutic utility (reviewed, e.g., in^4,5,68–70^). Rod phototransduction, with PDE6 as a key effector enzyme, is probably the best-known G-protein mediated signaling cascade in vertebrates, and PDE6 function and inhibition can be investigated electrophysiologically in live photoreceptors.

In this work, we utilized the possibility to obtain quantitative information on both basal and light-activated PDE6 as well as on their inhibition by the membrane-permeant non-selective PDE inhibitor IBMX in functional mouse rods. We found that despite the inhibition constant of IBMX being approximately the same for the basally activated and the light-activated PDE6 forms in normal calcium, lowering of Ca^2+^ changed both *K*_*I*_ values substantially, and to opposite directions: The *K*_*I*_ against basally activated PDE6 increased by more than three-fold while the *K*_*I*_ against light-activated PDE6 was approximately halved by decreasing Ca^2+^.

There are two kinds of explanations on how Ca^2+^ could modify the inhibition constant of IBMX against PDE6: either Ca^2+^ interacts directly with IBMX or directly or indirectly with PDE6. To our knowledge, there is no evidence of substantial interaction between IBMX and Ca^2+^, and additionally, our results suggest that Ca^2+^ can modify the basal PDE6 activity. Hence, we consider the latter option more probable.

Most phosphodiesterases share highly conserved amino acid sequences in their catalytic domains, suggesting high similarity in the hydrolytic sites between different PDEs^2,71–73^. IBMX is a small, non-selective competitive inhibitor that shares common binding sites with cGMP in the sub-pocket of the PDE catalytic site^71,74–76^. The observation that the *K*_*I*_ values for IBMX against basally activated and light-activated PDE6 are similar in normal calcium suggest that inhibition of both these PDE6 forms by IBMX are mediated through a common process, e.g., by direct competition by IBMX and cGMP. However, the differing effect of low Ca^2+^ on *K*_*I*_ values indicates more independent inhibition mechanisms for the basally activated and the light-activated PDE6. This complex inhibition would require that, in addition to competition between cGMP and IBMX for the catalytic site, the PDE6γ-subunit could interact with IBMX (directly or indirectly) and that this interaction would be modified by Ca^2+^ without significant effect on the catalytic activity of PDE6. Antagonistic interaction between the γ-subunit and IBMX would manifest as apparently larger inhibition constants determined in the presence of γ-subunits compared to those determined in the absence of γ-subunits, i.e. in trypsin-activated PDE6 (note the *K*_*I,light,normal Ca*_^*2+*^ = 13.8 µM determined here and the *K*_*I,trypsin*_ = 4.5 µM determined in^48^). In order to explain the low Ca^2+^-induced modulation of *K*_*I*_ values, the ability of the γ-subunit to hinder PDE6 inhibition by IBMX should weaken in the light-activated state and intensify in the basally activated state in low Ca^2+^. However, clarification of this mechanism requires further studies.

Our finding may also relate to a recent result suggesting that substantial PDE6 activation requires the binding of two transducins to one PDE6 dimer, the binding of the first transducin to the high-affinity transducin-binding site causing negligible cGMP hydrolytic activity while binding of the second transducin to the low-affinity transducin-binding site brings about the full PDE6 activity^77,78^. If the basal PDE6 activity was caused by the subunit containing the high-affinity transducin-binding site (without or with the binding of transducin) and the light-activated cGMP hydrolytic activity was produced by the subunit with the low-affinity transducin-binding site, the inhibition of these subunits by IBMX could differ. This might explain the different *K*_*I*_ against basally and light-activated PDE6 in low Ca^2+^.

## Data Availability

The datasets generated during and/or analysed during the current study are available from the corresponding author on reasonable request.
